# Chronic Wounds and Employment: Assessing Occupation‐Related Burden of Patients With Chronic Wounds—Results of a Pilot Study

**DOI:** 10.1111/iwj.70372

**Published:** 2025-04-16

**Authors:** Dorothee Ann‐Kathrin Busch, Nicole Methner, Delara Azodanlou, Maurice Moelleken, Joachim Dissemond, Ursula Hertha Hübner, Mareike Przysucha, Florian Kücking, Carola Berking, Cornelia Erfurt‐Berge

**Affiliations:** ^1^ Department of Dermatology University Hospital Erlangen, Friedrich‐Alexander‐Universität Erlangen‐Nürnberg Erlangen Germany; ^2^ Health Informatics Research Group, Osnabrück University of AS Osnabrück Germany; ^3^ Chair of Social Psychology Friedrich‐Alexander‐Universität Erlangen‐Nürnberg Erlangen Germany; ^4^ Department of Dermatology, Venerology and Allergology University Hospital of Essen Essen Germany

**Keywords:** chronic wounds, employment, quality of life, work, wound healing

## Abstract

Chronic wounds can impact the quality of life of working‐age individuals. However, the specific challenges and burdens these patients face in the workplace remain understudied. This study aimed to 1) investigate how chronic wounds affect work life and 2) develop a screening tool for identifying highly affected patients. In total, 51 patients with chronic wounds answered a questionnaire on demographics, employment status, wound‐related limitations, workplace conditions, social welfare use and subjective burden of disease. To assess the subjective burden, we developed a specific research tool on employment and chronic wounds (REACH Score) to measure and quantify the impact of the chronic wound on working patients. The patients, who answered the questionnaire, were employed (78%), on sick leave (18%) or retired (4%). They spent an average of 5.3 h per week on wound‐related activities. Regarding workplace stressors, we found that taking breaks when needed was correlated with less time off sick and better work‐related quality of life. Patients reported career concerns and reduced productivity. The REACH score was significantly correlated with sick leave, work difficulties, time consumed by the wound and overall quality of life. According to our pilot‐study, the impact of chronic wounds on patients of working age is most evident in the form of sick leave and reduced work performance and in a reduced quality of life. It is crucial to identify the key factors contributing to stress in the work environment in a larger sample in order to improve the working conditions of patients and detrimental socioeconomic effects on the workforce. The REACH score is a novel tool to screen employed patients with chronic wounds for reduced work capacity and quality of life.

**Summary**

- Chronic wounds significantly impact the work lives of individuals of working age. This includes increased sick leave, reduced productivity, and diminished quality of life.
- Workplace factors contribute to stress and impact the well‐being of patients with chronic wounds. Identifying and addressing these factors is crucial for improving working conditions and supporting these individuals.
- The newly developed REACH score is a valuable tool for screening employed patients with chronic wounds for these adverse effects. It can help identify individuals at risk and guide targeted interventions.

## 1 Introduction

Chronic wounds affect approximately 2.5% of the US population, imposing burdens on both patients and healthcare systems [1]. They often lead to reduced quality of life and participation, mainly due to severe pain, impaired mobility, and emotional distress [2, 3, 4, 5]. Most patients are elderly, on average 70–80 years [6]. However, younger individuals, who are often still part of the workforce, are also affected [7]. A cross‐sectional study in Germany that included information on employment showed that 9% of patients with wounds were employed [8].

Socio‐psychological aspects and patient‐reported outcomes are increasingly relevant to the management of patients with wounds [9, 10, 11]. Tools to measure disease‐specific, health‐related quality of life such as the Wound‐QoL are well established in this field [9, 11, 12]. The general reduction of quality in life in patients with wounds has been measured repeatedly [2, 13, 14, 15]. In addition to the personal burden chronic wounds pose for working patients, there is a significant socioeconomic dimension including indirect costs resulting from reduced work performance and dependence on the social welfare system [8, 16].

The social welfare system in Germany offers various support options for those unable to work, including paid sick leave and additional measures that can be applied for, such as rehabilitation or disability pensions. Medical facilities, charities, and government institutions offer the opportunity to obtain advantage of socio‐medical advice.

To our knowledge, it has not been shown whether these measures are effective in supporting patients who experience restrictions in their work due to chronic wounds.

As far as the individual level is concerned, employed patients with chronic wounds face significant challenges. Reduced participation and social isolation have been described [5, 17].

Studies on the employment situation of patients with chronic wounds are rare, but some qualitative research highlights significant work‐related challenges. For instance, Da Silva et al. [18] conducted a semi‐structured interview study with 14 patients, which highlighted work‐related difficulties, such as financial issues and having to withdraw from work. Other qualitative studies, one with eight men with venous leg ulcers and one interview study with 14 patients with diabetic foot ulcers, reported patients stopping work due to chronic wounds [19, 20].

In the patients with diabetic foot ulcers nearly half experienced early retirement due to job loss [20]. Reviews confirm that chronic wounds often reduce work capacity and lead to premature retirement [21, 22, 23, 24, 25, 26]. Despite these findings, there is a lack of quantitative data on the nature and extent of the challenges employed individuals with chronic wounds face.

Consequently, to effectively address these challenges, a comprehensive understanding of how chronic wounds and employment influence one another is essential.

The present study aimed to answer the following questions:

- How do chronic wounds impact work performance?
- Are affected individuals burdened?
- What factors cause the wound to become a burden to working patients?
- How can particularly burdened patients be identified?

To answer these questions, we aimed to develop a screening tool to assess wound‐related limitations in employed patients and conduct a pilot study to collect initial data. This will help to identify opportunities for supportive measures and improve patient care.

## 2 Materials and Methods

### 2.1 Development of a Specific Questionnaire for Employed Patients With Chronic Wounds

In a multi‐professional team of wound experts, dermatologists, and psychologists, we developed a 50‐item questionnaire for employed patients with chronic wounds accompanied by a 17‐item research tool on employment and chronic wounds (REACH) Score to assess wound‐related restrictions at work. The questionnaire was developed through an iterative process involving multiple experts, including wound care specialists from both medical and nursing backgrounds, an expert in occupational dermatology, and methodological input from Author 2. A qualitative comprehensibility analysis was conducted with 5 participants, after which minor changes to phrasing and question formats were made to enhance clarity.

Completion, including consent, took around 20 min, and physicians provided additional data on diagnosis, wound location and size, pain (0–10 numeric rating scale), and Wound‐QoL.

The 50‐item questionnaire comprises six parts:

- Epidemiological and clinical data: Including education, marital status and duration of the chronic wound.
- Wound‐related limitations: Time spent on wound‐related activities, reduction of working hours.
- Occupation and occupational status: Professional qualification, current occupation, weekly working hours, sick leave.
- Current conditions at work: Sitting or standing work position, physical labour, working in cold or hot conditions, possibility to take breaks as required
- Use of the social welfare system: Socio‐medical advice, disability pension, or rehabilitation measures.
- Subjective burden of disease: The REACH Score and a visual analogue scale (VAS).

### 2.2 The REACH Score

In order to capture the subjective, occupation‐related burden of the wound in a nuanced way, we developed the REACH Score, which was created with reference to the existing Wound‐QoL [9] and Erlangen Life Quality Assessment for wound associated relatives (“ELWA”) questionnaire, which is used to assess quality of life in family members of patients with chronic wounds [27]. The REACH Score includes statements on the occupational impacts of chronic wounds, rated by respondents on a Likert scale with four levels of agreement: 1 = *completely inapplicable*, 2 = *rather inapplicable*, 3 = *rather applicable*, and 4 = *completely applicable*. The score consists of 17 items with statements about limitations and covers various aspects of life including leisure and recreation, absence and illness at work, worry and dejection, lack of cognition or sleep, worries about the future, support or pressure in the work environment, and the need for socio‐medical counselling (refer to Table A1). The score ranges from 1 to 4 (mean score; sum score: 17–68) and is calculated from the unweighted mean score of the responses. A higher score corresponds with a higher burden of the chronic wound in the context of occupation. To identify the patients that were most burdened, we calculated quartiles.

### 2.3 Patient Sample

A chronic wound (non‐healing after 8 weeks), age under 65 years, and either current or previous employment with a florid chronic wound were inclusion criteria for the study. Patients under 18 years, patients who were unable to adequately read and understand the questionnaire, and patients who filled in less than half of the questionnaire were excluded.

### 2.4 Data Collection and Evaluation

Clinical information about wound size, wound cause, relevant further diagnoses, pain, and Wound‐QoL were extracted from the patients' routine data chart. The questionnaire was distributed between 01.01.2022 and 31.12.2023 at the specialised wound centres of two university hospitals. Participation in the survey was voluntary after appropriate informed consent.

### 2.5 Statistical Analyses

Analyses were performed using IBM (New York, USA) SPSS statistics version 29 and R 4.4.1. Not all patients completed the entire questionnaire. Therefore, we present frequencies that refer to the sum of all valid responses (i.e., without missing values). To determine statistical relationships, we calculated Pearson's correlation coefficients, unless otherwise stated. To analyse differences between groups, we calculated Mann–Whitney‐U‐tests. Effect sizes are interpreted according to Cohen as follows: *r* = 0.01 as small, *r* = 0.30 as medium, and *r* = 0.50 as large [28].

## 3 Results

### 3.1 Patient Sample

A total of 55 patients answered the questionnaire during their routine appointments. Four patients were excluded because either the consent form was not fully completed or the patients did not work while the chronic wound was present. This left a total of 51 patients whose questionnaires were analysed (for characteristics of the patient population, Tables 1 and 2).

**TABLE 1:** Description of patient population.

	*n*	%
Gender		
Men	27	52.9
Women	24	47.1
Gender diverse	0	0
Occupation		
Employed	39	76.4
Currently on sick leave	7	13.7
Reduced earning capacity	4	7.8
Other	1	2.0
Main diagnosis		
Venous leg ulcer	17	33.3
Pyoderma gangrenosum	15	29.4
Arteriovenous leg ulcer	4	7.8
Post‐traumatic wound	1	2.0
Other	14	27.5
Wound size in cm^2^		
< 30	33	64.7
30–100	9	17.6
101–200	4	7.8
> 200	5	9.8

**TABLE 2:** Patient population: Age, school years, and other clinical data.

	*n*	*M* (SD)
Age in years	51	49.4 (12.0)
School years	44	11.0 (1.8)
Duration of the current chronic wound in months	50	18.9 (28.4)
Pain (NRS) at rest	40	2.3 (2.6)
Pain (NRS) during dressing changes	29	3.8 (3.0)

## 4 How Do Chronic Wounds Impact Work Performance?

### 4.1 Reduction in Work Performance

Chronic wounds resulted in a reduction in work performance, which was mainly noticeable in the form of sick leave. Of the 51 patients, 76.4% (40) of patients were currently employed, while 13.7% (7) were on sick leave and 7.8% (4) had a reduced earning capacity.

Overall 68.6% (35) of patients were or had been on sick leave due to chronic wound. 14 patients (27.5%) reported having been on sick leave more than three times in total. Four patients (7.8%) reduced their weekly working hours, and two patients (3.9%) were dismissed from their work because of the wound.

### 4.2 Utilisation of the Social Welfare System

We aimed to determine if patients benefited from the German social welfare system's support options. Among 51 patients, nine (17.6%) applied for social support. The most common support was occupational rehabilitation (six patients, 11.8%), followed by a disability pension (five patients, 9.8%) (multiple answers were possible). Four (7.8%) patients consulted their company doctor regarding their chronic wound.

Only one patient (2.0%) received socio‐medical counselling, although seven patients expressed an interest in it. Nine patients (17.6%) were unaware of counselling options, and two‐thirds of whom expressed interest. More than half of the patients (33 patients, 64.7%) reported no need for counselling.

## 5 Are Affected Individuals Burdened?

### 5.1 REACH Score

The REACH Score measures the subjective restrictions in working life of patients with a chronic wound. Regarding the reliability of the score, we found a high internal consistency (Cronbach's α 0.90).

**FIGURE 1:**
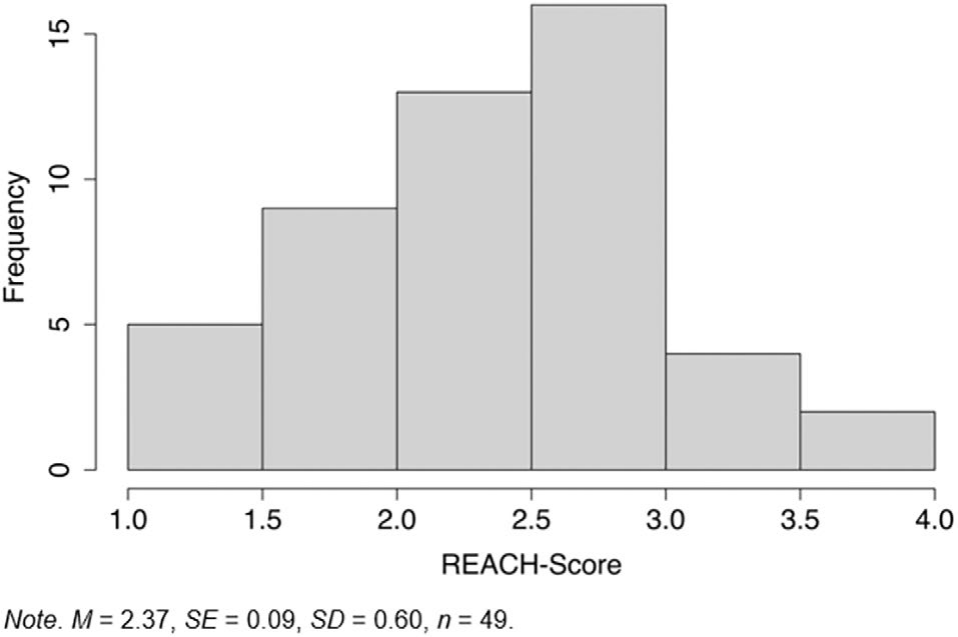
Distribution of the REACH Score.

The mean score ranges from 1 to 4 (accordingly, the sum score ranges from 17 to 68). Overall, the sample reported a moderate impairment (*M* = 2.37, SD = 0.60). Around 55.1% of patients did not feel very burdened with the chronic wound in terms of their employment (a score lower than the scale midpoint of 2.5), whilst 44.9% of the patients perceived the wound as a burden (a score higher than 2.5; see also Figure 1).

When looking at individual items of the REACH Score, some results stand out:

60.4% felt that the wound affected them negatively at their work.

Approximately 66.0% of patients were worried about the wound and their future career. 67.4% felt less productive and 79.6% of patients felt that the wound had a negative impact on their leisure time.

### 5.2 Indicators of Validity

#### 5.2.1 VAS Score

**FIGURE 2:**
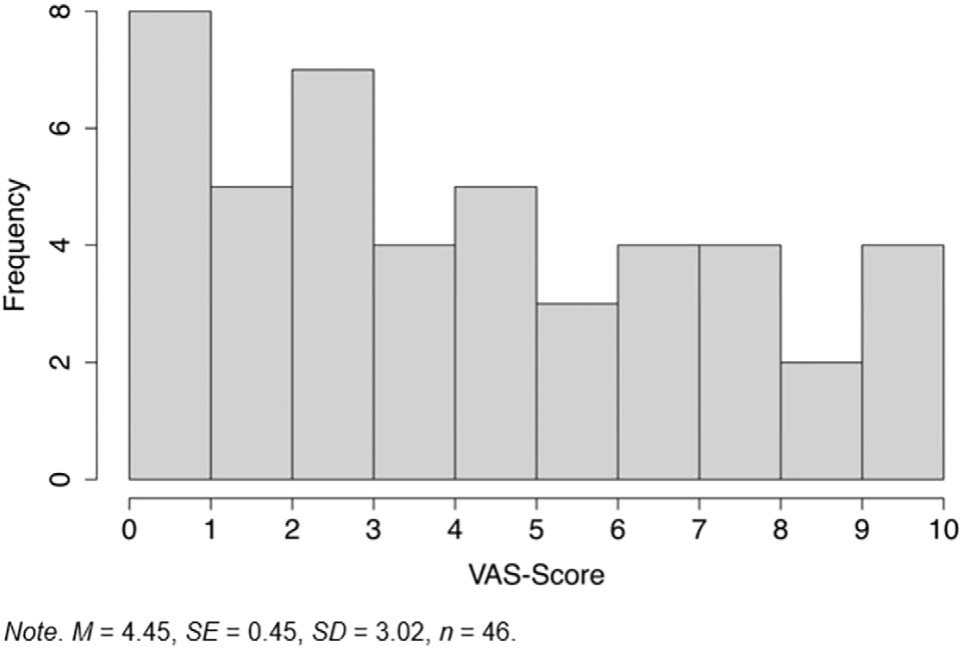
Distribution of the VAS Score.

Patients were asked to indicate on a visual analogue scale, ranging from 0 to 10, the extent to which they felt restricted in their professional activities due to the wound. The responses were widely distributed and reflected the results of the REACH Score (see Figure 2). With regard to the subjective stress caused by the wound and the occupational activity, the REACH Score and VAS Score were strongly correlated, as expected (Table 3).

**TABLE 3:** Correlations for main study variables.

Variables	1	2	3	4	5	6	7	8	9	10	11
1. Duration of the chronic wound	—										
2. Wound size (< 30, 30–100, 101–200, > 200 cm^2^)^a^	0.05 [−0.24, 0.33]	—									
3. Diagnosis leg ulcer (1 = yes, 0 = other)	0.20 [−0.09, 0.45]	0.16 [−0.13, 0.42]	—								
4. Pain (NRS) at rest	0.25 [−0.08, 0.52]	0.03 [−0.29, 0.35]	0.28 [−0.04, 0.54]	—							
5. Pain (NRS) during dressing changes	0.29 [−0.09, 0.60]	0.28 [−0.11, 0.59]	0.35 [−0.02, 63]	0.70 [0.45, 0.85]**	—						
6. Subjective occupational burden of disease (REACH Score)	0.06 [−0.23, 0.34]	0.07 [−0.22, 0.35]	−0.08 [−0.35, 0.21]	0.22 [−0.11, 51]	0.35 [−0.02, 0.64]	—					
7. Perceived restriction of professional activity due to wound (VAS Score)	0.13 [−0.17, 0.40]	0.09 [−0.21, 0.38]	−0.17 [−0.44, 0.13]	−0.01 [−0.33, 0.32]	0.25 [−0.14, 0.56]	0.75 [0.59, 0.85]**	—				
8. Health‐related quality of life (Wound‐Qol)	0.42 [0.10, 0.67]*	0.11 [−0.24, 0.44]	0.12 [−0.22, 0.43]	0.58 [0.28, 78]**	0.77 [0.47, 0.91]**	0.50 [0.19, 0.72]*	0.35 [−0.01, 0.53]	—			
9. Time consuming activities because of the wound	0.06 [−0.23, 0.33]	0.29 [0.01, 0.53]*	0.01 [−0.27, 0.28]	0.25 [−0.06, 52]	0.53 [0.20, 0.75]*	0.54 [0.31, 0.72]*	0.47 [0.20, 0.67]**	0.43 [0.11, 0.67]*	—		
10. Challenges for commute (0 = no, 1 = yes)	−0.27 [−0.55, 0.06]	0.02 [−0.31, 0.35]	0.05 [−0.23, 0.37]	−0.14 [−0.49, 0.26]	0.06 [−0.39, 0.49]	0.38 [0.06, 0.63]*	0.36 [0.04, 0.62]*	0.10 [−0.31, 0.48]	0.24 [−0.09, 0.52]	—	
11. Sick leave (0 = no, 1 = yes)	−0.03 [−0.31, 0.26]	0.03 [−0.27, 0.31]	0.05 [−0.23, 0.33]	0.16 [−0.17, 0.45]	−0.02 [−0.40, 0.36]	0.34 [0.06, 0.57]*	0.25 [−0.05, 0.51]	−0.01 [−0.35, 33]	0.04 [−0.24, 0.32]	0.29 [−0.04, 0.56]	—
12. Application for disability pension or rehabilitation measures (0 = no, 1 = yes)	−0.01 [−0.28, 0.28]	−0.06 [−0.34, 0.23]	−0.02 [−0.30, 0.26]	−0.04 [−0.35, 0.29]	0.05 [−0.34, 0.42]	0.29 [0.01, 0.53]*	0.25 [−0.05, 0.50]	0.06 [−0.29, 0.40]	0.14 [−0.15, 0.40]	−0.11 [−0.42, 0.22]	0.10 [−0.19, 0.37]

*Note:* 95% confidence intervals are in [brackets].

^a^ Spearman rank correlation.

* *p* < 0.05.

** *p* < 0.001.

#### 5.2.2 Sick Leave

Patients were asked whether they had been off work due to the chronic wound in the previous 12 months. Two thirds of our sample were or had been on sick leave. If the REACH Score is valid, it should be related to sick leave. Indeed, patients who were or had been on sick leave also reported higher REACH Scores (*M* = 2.52, SD = 0.58) than those who have not been on sick leave (*M* = 2.08, SD = 0.49), U(n1 = 34, n2 = 14) = 350.50, z = 2.55, *p* = 0.011, *r* = 0.34, indicating a moderate relation between REACH Score and sick leave, hence further supporting the score's validity.

#### 5.2.3 Subjective Burden of Disease

Chronic wounds had a negative impact on patients' quality of life (Table 3). To measure the Health‐Related Quality of Life for Chronic Wounds, we used the Wound‐QoL, which was strongly correlated with the REACH Score (*r* = 0.50, *p* < 0.01). However, unlike the REACH Score, the Wound‐QoL was not correlated with sick leave or the use of the welfare system. The Wound‐QoL was correlated with pain at rest, wound duration, and time‐consuming activities.

## 6 Which Factors Cause the Wound to Become a Burden to Working Patients?

### 6.1 Factors Affecting Subjective Burden

There were no significant correlations between the subjective, occupation‐related burden of disease (i.e., the REACH Score) and clinical data, e.g. wound size or pain at rest (NRS). Instead, the subjective, occupation‐related burden of disease was related to everyday stresses due to the wound, namely, time‐consuming activities like dressing changes and challenges for the journey to work (Table 3).

### 6.2 Time Consuming Activities Related to the Wound

Patients with chronic wounds spent an average of 5.3 h per week on wound‐related activities (Table 4). Dressing changes were the most time‐consuming, taking an average of 2.7 h per week. The additional time spent on wound care was correlated with the REACH Score, indicating a higher subjective burden of disease (Table 3).

**TABLE 4:** Time consuming activities because of the chronic wound.

Wound‐related activities	*n*	*M* (SD) hours per week	*Min–Max* hours per week
Dressing changes	50	2.7 (2.9)	0.0*–*14.0
Restrictions in personal hygiene	50	0.8 (1.6)	0.0*–*7.0
Additional household chores (disinfection, laundry)	51	0.7 (1.8)	0.0*–*10.0
Doctor's appointments	51	1.1 (1.2)	0.0*–*4.0
Total	51	5.3 (5.6)	0.0*–*23.0

### 6.3 Challenges for the Journey to Work

One third of the patients reported difficulties commuting to work or having to change their mode of transportation due to the chronic wound, for example having to take the car instead of the bike to get to work or not being able to walk longer distances anymore. Having difficulties getting to work was also correlated with a higher REACH Score (Table 3).

### 6.4 Working Environment

We asked about the patients' work circumstances to identify potential factors contributing to the wound becoming a burden (Table 5).

**TABLE 5:** Working conditions.

Work conditions	%
Working while standing	37.3
Working while sitting	39.2
Walking	41.2
Crouching/kneeling	5.9
Lots of physical exertion	21.6
Changing body positions	49.0
High temperatures	13.7
Cold temperatures	7.8
Elevating the leg affected by the wound	5.9
Breaks as required (e.g., for pain)	37.3
Having informed the coworkers about the restrictions due to the wound	70.6

*Note:* Each patient could check several options.

This information could help to better adapt the professional environment. Interestingly, we only found that being able to take breaks was correlated with a lower REACH Score, *r*(47) = −0.30, 95% CI[−0.54–0.01], *p* = 0.04, and with less sick leave, *r*(47) = −0.32, 95% CI[−0.55, –0.03], *p* = 0.03.

## 7 How Can Particularly Burdened Patients be Identified?

### 7.1 REACH Score as a Screening Tool

Overall, some patients felt that their chronic wound was a significant burden on their work life (55.1% according to the REACH score), while others reported fewer challenges (44.9%; see also Figures 1 and 2). Therefore, only some patients may benefit from supportive measures such as socio‐medical counselling. Based on our pilot sample, we aimed to identify a cut‐off value of the REACH Score that would indicate the most burdened individuals. This could provide a first indication of which patients should be recommended for socio‐medical counselling.

To identify a cut‐off value based on our sample, we used quartiles (rather than percentile ranks which may be used for larger samples; as recommended by Goldhammer and Hartig [29]). Patients were ranked according to their REACH Scores and the sample was divided into four equally sized groups (i.e., quartiles). Thus, the first quartile represents 25% of the patients reporting the lowest burden, while the fourth quartile represents the quarter reporting the highest burden.

We suggest recommending socio‐medical counselling for patients with the highest burden; thus, patients in the fourth quartile (Table 6). Accordingly, the cut‐off value is a REACH Score of 2.76 (based on the REACH mean score; correspondingly, for a sum score, the cut‐off value is 46). In short, based on the current data, we suggest recommending socio‐medical counselling to patients with a REACH Score of 2.76 or higher.

**TABLE 6:** Quartiles and the corresponding REACH scores.

Quartil	%	REACH score (mean score)	REACH score (sum score)^a^	Interpretation
Q1	≤ 25	1.00–1.88	17–29.50	Least burdened quarter
Q2	−50	−2.35	39.00	Second least burdened quarter
Q3	−75	−2.76	46.00	Second most burdened quarter
Q4	−100	−4.00	68.00	Most burdened quarter

*Note:* Q2 equals median. *n* = 49.

^a^ If there are missing data (i.e., not all 17 items of the REACH Score were answered), the sum score should not be interpreted; instead, we recommend using the mean score.

## 8 Discussion

Our study explored the interaction between chronic wounds and employment. For the first time, we collected comprehensive data on work‐life experiences in the context of chronic wounds, regardless of whether patients felt burdened or not, and developed a screening tool for this specific patient population. Our findings revealed significant limitations in both work life and personal life for patients with chronic wounds. Some of the highest scoring factors limiting patients were having their leisure time (e.g., hobbies, friends, holiday) affected by the chronic wound, being worried about their professional future, feeling less efficient at work, and feeling less refreshed after weekends, holidays, or time off work. The impact of chronic wounds on professional life was particularly evident in reduced work performance seen in increased sick leave and, in some cases, premature retirement.

To measure patients' subjective burden of disease related to their chronic wounds, we developed the REACH Score and employed the VAS Score. Overall, patients differed in their perceived burden. Not all patients felt their chronic wounds interfered with their work. The restrictions imposed by chronic wounds varied, with 45% reporting being affected in their work. However, this does not necessarily imply that the remaining patients were not significantly affected. Interestingly, a higher percentage of patients (79.6%) reported that their leisure time was impacted by the chronic wound. Chase et al. found similar results in a descriptive analysis of 21 patients with chronic venous ulcers. They observed reduced capacity for both work and leisure activities, despite the patients generally perceiving themselves as relatively healthy [10, 30].

A key objective of this study is to identify how to alleviate the burden of chronic wounds. By examining the circumstances contributing to increased strain on the patient, we can identify potential strategies for reducing this burden. While well‐known wound‐specific factors like size, pain, and duration remain crucial, additional considerations are necessary for employed patients with chronic wounds. One factor that seemed to play an important role was the ability to take breaks at work. This was also correlated with fewer sick days. So, taking breaks when needed seems to be a possible approach to reducing stress at work and the associated economic loss. Furthermore, reducing time‐consuming wound‐related activities may significantly benefit this patient group. Self‐management interventions could be a focus, empowering patients to manage their condition effectively and potentially save time [31]. Despite the restrictions, burden, and number of sick days because of their wound, the support options available through the German social welfare system were relatively underutilised. Our research suggests that a greater proportion of patients would benefit from socio‐medical counselling, for example, for support in applications for rehabilitation measures or for reduced earning capacity.

Given that not all patients are equally burdened, it is essential to assess the individual burden of each patient and identify the most distressed patients to target support. The VAS Score could easily be incorporated into medical history forms, and the REACH Score offers a simple way to capture occupational stress in a more nuanced way.

To evaluate the REACH Score's validity in measuring job‐related burden from chronic wounds, we analysed its correlation with wound‐related stress indicators like the VAS and sick leave frequency. Our findings support the score's validity.

Furthermore, the REACH Score was correlated with the established Wound‐QoL. While the Wound‐QoL assessed general limitations caused by chronic wounds, the REACH Score appeared to be more specific in identifying work‐related declines in quality of life. In general, disease‐specific domains should be assessed in addition to general factors affecting quality of life [32], hence the development of a measure to screen for wound‐specific impairment is crucial. The REACH Score offers promising possibilities for screening in this particular patient population, given the limited data on the interaction between chronic wounds and occupation. Based on the present data, quartiles can be used to provide support to the most vulnerable patients. Depending on the availability of socio‐medical counselling, the cut‐off value can be adjusted accordingly.

### 8.1 Limitations

The present pilot study has limitations due to the bias of university institutions. Rare and inflammatory causes like pyoderma gangrenosum were found more frequently than in the general population with chronic wounds [33].

Due to the small size, some correlations may be significant by chance, and some may not be significant even though there is a relation between the variables [34]. Moreover, correlations allow no conclusions about causality. Potential strategies to change subjective burden should be interpreted as preliminary.

Our aim is to validate the REACH questionnaire for use in future research. The project is still ongoing, and adding more centres, involving other healthcare providers, and performing psychometric validation on the questionnaire are future goals. By increasing the sample size, more comprehensive data can be collected. Future research should also explore the influence of job type on distress and collect more detailed data on occupational categories.

## 9 Conclusions

Our study comprehensively investigated the work‐related circumstances and challenges faced by 51 patients with chronic wounds. We found that a primary indicator of reduced work performance is increased sick leave, while other social support options are rarely used. Time‐consuming activities, such as dressing changes and doctor's appointments, and commuting difficulties negatively affect employed patients, while the ability to take breaks in the work environment appears to be a critical factor in reducing patient burden.

The REACH Score is a promising tool for measuring the burden of occupational patients with chronic wounds and represents an additional and potentially more specific tool for this subgroup. Approximately 45% of patients reported a negative impact of their wounds on their work life, while approximately 80% were affected in their leisure time. These findings highlight the importance of screening for distress in this patient group and offering support, such as socio‐medical counselling, to those requiring it.

Future research should explore the influence of job type on distress and collect more detailed data on occupational categories.

## Ethics Statement

The Ethics Committee approved the implementation of the study (Vote Nr. 22‐27‐B).

## Conflicts of Interest

The authors declare no conflicts of interest.

## Data Availability

The data is available from the authors upon reasonable request.

## Appendix A

**TABLE A1:** Questionnaire to calculate the REACH Score.

In the last 3 months …	Completely inapplicable	Rather inappliclable	Rather applicable	Completely applicable
The wound affects me at my work	O	O	O	O
My leisure time suffers from the chronic wounds	O	O	O	O
I feel less refreshed after weekends, holidays or time off work	O	O	O	O
I have difficulty sleeping	O	O	O	O
I often feel ill	O	O	O	O
I call in sick more often	O	O	O	O
I actually am sick more often	O	O	O	O
I have more wound pain at work than at home	O	O	O	O
I feel unfocused at work	O	O	O	O
I feel depressed and sad	O	O	O	O
I am very worried about my chronic wound and my professional future	O	O	O	O
I do not feel as professionally productive as I used to be	O	O	O	O
I would like more advice on socio‐medical issues	O	O	O	O
I have feelings of guilt towards my colleagues/employer because I am not fully productive	O	O	O	O
My colleagues and superiors are understanding that I am restricted because of my wound. (*recoded*)	O	O	O	O
My employer is placing pressure on me because of the chronic wound	O	O	O	O
I am afraid of being dismissed because of my chronic woun	O	O	O	O
